# Greater Food-Related Stroop Interference Following Behavioral Weight Loss Intervention

**DOI:** 10.4172/2165-7904.1000187

**Published:** 2013-08-28

**Authors:** Kathryn E Demos, Jeanne M McCaffery, Sara A Cournoyer, Caroline A Wunsch, Rena R Wing

**Affiliations:** Weight Control and Diabetes Research Center, Department of Psychiatry & Human Behavior, The Miriam Hospital and Warren Alpert Medical School of Brown University, USA

**Keywords:** Food-stroop paradigm, Weight loss intervention, SWLM

## Abstract

**Objective:**

Individuals who have successfully lost and maintained weight have slower reaction times on food-related Stroop tasks, indicating greater cognitive interference to food stimuli compared to obese and normal weight individuals. It remains unclear whether this interference is a preexisting characteristic of weight loss maintainers or if food-interference changes in obese individuals as they lose weight.

**Method:**

To examine potential changes in food-related interference, a food-Stroop paradigm was used to measure responses to food versus non-food words in 13 obese women before and after a 12-week behavioral weight loss program.

**Results:**

Participants achieved a mean weight loss of 5.12 kg through the behavioral weight loss program. Their reaction time to food words became significantly slower (p<0.001) and they made significantly more errors (p<0.01) following treatment.

**Discussion:**

These findings suggest that through behavioral weight loss treatment obese individuals experience increased interference toward food words, which may reflect increased salience of food-related cues. Future research is needed to determine whether increases in interference are related to better weight loss and maintenance.

## Introduction

A number of studies have used the Stroop paradigm to investigate cognitive processing, specifically food-related processing, in patients with eating disorders. The standard color-word Stroop task is a measure of cognitive interference designed to quantify the time required to inhibit a prepotent response, like word reading, in order to attend to and perform a less dominant task, such as identifying text color [1]. To assess food-related interference, researchers have adopted this traditional Stroop task by including food words [2–4]. Historically, a disorder-salient Stroop effect has been observed, wherein individuals with eating disorders show greater interference (i.e., are slower to respond) for disorder-relevant words [5].

With increasing rates of obesity and its associated co-morbidities, more emphasis has been placed recently on investigating the cognitive processes associated with obesity and successful weight loss and maintenance. Previous studies show that successful weight loss maintainers (SWLM) self-report high levels of cognitive restraint [6,7], and exhibit increased activity in brain regions associated with cognitive control when viewing food images [8]. Recently, Phelan and colleagues compared responses of obese, normal weight, and SWLM to a food-related Stroop task [4]. They found significantly greater interference, including slower reaction time and more errors, for high-calorie food words in the SWLM compared to both obese and normal weight individuals [4]. This finding is interpreted as greater cognitive processing bias towards high-calorie food cues, which may reflect the ongoing cognitive control these individuals must exert in order to maintain their weight loss [4].

Although highly informative, these studies have been cross-sectional in nature, and therefore do not provide insight into whether cognitive processes change as a function of weight loss or whether there are pre-existing group differences. Therefore we sought to extend these findings by examining food Stroop interference in obese women before and after a 12-week behavioral weight loss intervention. Based on this previous work, it was hypothesized that participants would exhibit greater interference to food words (i.e., slower reaction times) posttreatment compared to pre-treatment.

## Methods

### Participants

Fourteen obese women (BMI 30.0 – 40.0, mean age=49.9) were recruited via self-referral to participate in a 3-month behavioral weight loss intervention at the Weight Control and Diabetes Research Center in Providence, RI. One participant elected to not continue with the study following the initial baseline assessment and therefore data are reported for the remaining 13 participants. All participants were weight stable upon enrollment in the study (defined as within +/− 5 lbs. for the past two months). Recruitment for this cohort of participants began in March of 2010, and data collection for this group was complete in August of 2010.

### Ethics statement

This study was approved by the Internal Review Boards of The Miriam Hospital and Brown University. All participants provided written informed consent in accordance with The Miriam Hospital Internal Review Board and received monetary compensation for completing the assessments involved in the study.

### Procedures

Participants underwent a laboratory session just before the beginning of the clinical intervention (with a maximum lag of 30 days) and after the completion of the program (with a maximum lag of 30 days). Participants completed this session while in a fasted state (mean = 178.2 minutes, no difference pre- and post-treatment, p=0.29). During this session participants completed a food-related Stroop interference task that has been previously described [4]. Using E-Prime stimulus presentation software (Psychology Software Tools, Pittsburgh, PA), words from three categories (non-food, e.g., ‘chair’; low-calorie, e.g., ‘lettuce’; and high-calorie, e.g., ‘pizza’) matched on number of syllables were presented in blocks and displayed one at a time on a computer screen. Each target word was presented in red, blue, or green font. Participants were instructed to indicate the color in which the words were displayed via key press. Each block lasted 45 seconds and contained words from a single category. Trials within blocks were self-paced such that participants could complete as many trials in each block as possible. Responses and reaction times were recorded via E-Prime. Following the behavioral weight loss intervention (described below) participants returned for a second laboratory session and performed the same food Stroop task.

To account for the potential of global cognitive changes through treatment, participants completed a standard color-word Stroop (based upon the Golden paradigm and described previously by Phelan et al. [4]). An emotional Stroop that followed similar methods but included non-food words of neutral, negative, and positive valence (lists adapted from Larsen et al. [9] also included in order to assess whether responses to food words could simply be a function of food words taking on positive or negative valence. Subjects also completed demographic questionnaires and the Stunkard Three-Factor Eating Questionnaire [10].

### Intervention

Participants completed a 12-week group behavioral weight loss intervention consisting of weekly meetings incorporating diet, exercise, and behavioral therapy led by masters and doctorate level interventionists. All participants were placed on a standard caloric and fat restricted diet (e.g., 1200–1500 kcals/day and 30% fat, depending on initial weight) consistent with AHA, ADA, and ACSM recommendations [11–13], and received a fat/calorie guidebook and a food diary to record all food consumed and the corresponding calories and fat grams. Interventionists reviewed these diaries weekly and provided written feedback. All participants were encouraged to increase their physical activity gradually to at least 200 minutes per week using activities similar in intensity to brisk walking in bouts of at least 10 minutes.

### Analyses

Response times for correct food-word trials were analyzed using a repeated measure Analysis of Variance (ANOVA) with time (pretreatment vs. post-treatment) as the within-subject factor. To control for individual differences in psychomotor speed, response times to non-food words were statistically co-varied. The number of correct food-word trials was also analyzed using the same repeated measures ANOVA, with the number of correct non-food words statistically covaried. Analyses were conducted using SPSS software.

## Results

Participants lost an average of 5.12 ± 2.88 kg (Mean ± SD) with a range from 0.45–9.26 kg lost which corresponds to an average loss of 5.5 ± 3.4 percent of body weight (range=0.62%–11.4%). Correspondingly, participants reported increases in dietary restraint and decreases in disinhibition (Table 1).

The repeated measures ANOVA for reaction times (RTs) on the food-related Stroop revealed a significant effect of time (F[1, 13]=19.29, p=0.001), reflecting slower RTs (i.e., greater interference) at time 2(T2) relative to time 1(T1) (T1 adjusted mean=1053.1 ± 140.3 ms); T2=1062.2 ± 13.9 ms; Table 1). The effect of food type (high- versus low-calorie food words) and the interaction were not significant (all p’s > 0.05).

The repeated measures ANOVA for the number of correct trials on the food-related Stroop again revealed a significant effect of time (F[1, 13]=9.22, p=0.01). Participants responded correctly more often at T1 compared to T2 (T1 adjusted mean=44.5 ± 5.53 correct; T2=43.15 ± 4.98; Table 1). Neither the effect of food type nor the interaction was significant (all p’s >0.05). Thus despite slower reaction times, participants made significantly more errors post-treatment. Changes in RT and the number of correct trials on the food-related Stroop were not significantly correlated with changes in weight or percent body weight over this interval.

These effects were specific to the food-related Stroop. RTs to correct trials in the interference condition of the color-word Stroop (i.e., the incongruent condition, with word and color-matching conditions covaried) revealed no effect of time (p>0.05). Similarly, RTs to correct trials in the emotional Stroop also revealed no effect of time (p>0.05).

## Discussion

This is the first study to explore changes in cognitive interference for food words that occur over the course of behavioral weight loss treatment. We found that there were increases in cognitive interference to food words, as demonstrated by increased reaction time, in obese individuals after completing the 12-week weight loss program. Coupled with these increases in reaction time, participants demonstrated a decline in accuracy, with fewer correct trials post-treatment. It is important to note that both of these effects are contrary to expected practice effects (i.e., improved speed and accuracy with repeated task experience).

This study should be considered in the context of the literature on eating disorders and cognitive interference. Increased interference for food-related stimuli and body-image stimuli in individuals with bulimia nervosa (BN) and those with anorexia nervosa (AN) respectively has been shown repeatedly. Similarly, the obese individuals in the current prospective study exhibited increases in food-related interference over the course of a weight loss program. Taken together, these findings suggest that increased interference may stem from increased attention to food related stimuli, or increased salience of goal-relevant cues.

Previously, Phelan et al. demonstrated that SWLM also have greater interference albeit primarily for high calorie foods. In contrast, this study found no significant effects of food type. One possible explanation for this difference between SWLM and post-treatment obese individuals is that over time and practice these interference effects become more specific to food type, whereas immediately following a weight loss treatment program individuals have a broader cognitive bias that encompasses all foods.

Phelan and colleagues found no group differences in RT or errors during the standard color-word Stroop, suggesting that the response bias was specific to food stimuli. We extend these results by demonstrating that cognitive interference during the color-word Stroop does not change after behavioral weight loss intervention. Moreover, RT and error rates during the emotional Stroop did not differ before and after treatment, suggesting that the response bias to food words post-treatment does not simply reflect greater emotional reactivity.

Our results, taken together with the prior results of Phelan and colleagues, suggest that a cognitive processing bias towards food-related stimuli may be related to successful practices of weight loss strategies, either in individuals with long-term success with weight loss or those who have recently completed a weight loss program. It is plausible that practices such as self-monitoring and inhibiting high-calorie food intake associated with weight loss and maintenance require additional attention to food related stimuli, resulting in greater cognitive interference. Future studies are needed to explore the emerging relationship between food-related cognitive interference, weight loss and maintenance, and whether food-related cognitive interference may be altered to augment weight loss treatment outcomes.

## Figures and Tables

**Table 1:** Pre-and Post-Treatment measures of weight, dietary restraint, and performance on the food Stroop task.

Measure	Pre-Treatment	Post-Treatment	Mean Changes (Post – pre treatment)	p value
Weight (kg)	88.97 (13.53)	83.85 (13.53)	−5.12	0.0001
BMI	32.14 (2.90)	30.21 (3.18)	−1.93	0.0001
Restraint	7.67 (3.28)	13.08 (3.12)	5.41	0.0001
Disinhibition	10.17 (2.48)	7.25 (4.31)	−2.92	0.015
Hunger	8.25 (4.31)	5.33 (4.01)	−2.92	0.081
Reaction Time (ms; correct trials)^1^	1053.13 (140.25)	1062.23 (113.85)	9.10	0.001
Number Correct Trials^1^	44.50 (5.53)	43.15 (4.98)	−1.35	0.01

Mean values associated with pre- and post-treatment measures (standard deviations are presented in parentheses).

1 Response to high- and low-calorie food words combined, co-varying for responses to non-food words.
